# Photobiomodulation combined with dynamic thermal imaging as a diagnostic tool for chronic leg ulcers

**DOI:** 10.1007/s10103-026-04878-5

**Published:** 2026-04-29

**Authors:** Lilach Gavish, David Ben Daniel, Rita Lo, Sarah Olga Grinbaum, S. David Gertz, Oshrit Hoffer, Rose Raizman

**Affiliations:** 1 Institute for Research in Military Medicine (IRMM), Faculty of Medicine, The Hebrew University of Jerusalem and the Israel Defense Forces Medical Corps, Jerusalem, Israel; 2 The Department of Medical Neurobiology and The Saul and Joyce Brandman Cardiovascular Research Hub, Institute for Medical Research (IMRIC), Faculty of Medicine, The Hebrew University of Jerusalem, Jerusalem, Israel; 3 Department of Professional Practice, Scarborough Health Network, Centenary Hospital, Toronto, Canada; 4 Department of Electrical Engineering, Afeka Tel-Aviv Academic College of Engineering, Tel Aviv, Israel; 5 Lawrence S. Bloomberg Faculty of Nursing, University of Toronto, Toronto, Canada

**Keywords:** Thermography, Diabetic foot ulcers, Venous ulcers, Low level laser, Photobiomodulation, Microcirculation

## Abstract

Venous ulcers (VUs) and diabetic foot ulcers (DFUs) are the most common types of chronic leg ulcers but arise from distinctly different pathophysiologies. Photobiomodulation (PBM) has been shown to accelerate wound healing potentially through effects on microvascular perfusion. Chronic VUs respond less well to PBM therapy than DFUs. Previously, we showed a correlation between photobiomodulation-induced skin temperature changes and microvascular blood flow. The current study was designed to compare the acute microvascular response to photobiomodulation between venous and diabetic foot ulcers as assessed by dynamic thermal imaging. In this prospective study, 33 chronic wounds (22 DFUs, 11 VUs) were evaluated in 20 patients and treated with PBM (808 nm, 250mW peak-power, 15 kHz). Thermal images were captured before PBM, and every 5-minutes (min) thereafter, for 30 min. Minimum and maximum wound temperatures were recorded. Linear regression analysis was used to determine predictors of response. At baseline, wound bed temperatures (27.7 ± 2.9 °C) were significantly lower than the surrounding margins (difference of 4.6 ± 2.2 °C, *p* < 0.001) in both ulcer types. PBM treatment resulted in a sustained rise in wound bed temperatures in DFUs (ΔT = 2.10 ± 2.25 °C, *p* < 0.001) but not of VUs (ΔT = 0.68 ± 2.49 °C, *p* = 0.385). The thermal response to PBM of DFUs correlated inversely with baseline temperature (β=–0.521, *p* = 0.008; R²=0.399), indicating that colder DFUs showed the greatest response. These findings show that dynamic thermal imaging is useful for distinguishing between wounds likely, and those less likely, to benefit from photobiomodulation-induced stimulation of microvascular flow and may provide an important physiologically relevant diagnostic tool for patient-specific optimization of treatment.

## Introduction

Chronic leg ulcers are a major clinical challenge -- particularly among older adults. They can interfere significantly with the quality and expectancy of life and pose a substantial burden on health care delivery systems worldwide [1, 2]. Among chronic leg ulcers, venous ulcers (VUs) and diabetic foot ulcers (DFUs) are the most common; yet, they arise from distinctly different etiologies and pathophysiological mechanisms [1, 2]. Whereas the principal underlying abnormality in DFUs is less than adequate perfusion, prominent in the etiology of chronic VU is venous valvular insufficiency. Evidence has accumulated indicating that DFUs respond well to photobiomodulation (PBM) therapy [3–5], and enhanced microvascular blood flow has been proposed as the key underlying mechanism [6–8]. On the other hand, evidence of benefit of PBM for chronic VUs remains inconsistent with the majority of studies failing to demonstrate accelerated wound healing [9–19].

PBM involves non-ionizing optical radiation that modulates biological processes by interaction with endogenous chromophores through non-thermal, photophysical and photochemical events [20]. At the cellular level, PBM increases mitochondrial membrane potential [21, 22], promotes nitric oxide release [23], enhances proliferation and collagen synthesis in vascular smooth muscle cells [24], and reduces the expression and secretion of pro-inflammatory cytokines in activated macrophages [23]. At the tissue level, PBM promotes vascular dilation and angiogenesis [7, 25–27]. Clinically, PBM has been applied as a therapeutic modality to accelerate healing of hard-to-heal wounds [3–5, 9, 28, 29]. Enhanced microvascular blood flow has been proposed as a key underlying mechanism [8].

Thermal imaging is a non-invasive, passive, radiation-free technique for measuring surface temperature [30] by detection of infrared rays with its intensity converted to temperature values and displayed as color-coded images. A variety of physiological and pathological processes associated with changes in blood flow, such as inflammation, necrosis, or malignancy, can alter skin temperature and therefore be detected by this technique [31]. Dynamic thermal imaging involves tracking temperature changes over time in response to external challenges [32].

Beyond using PBM as a treatment modality, our group has pioneered its use as an experimental tool for assessing microvascular function. Previous studies from our group using dynamic thermal imaging of the hands of healthy volunteers showed that baseline skin temperature is a major determinant of the thermal response implicating microvascular regulation [7]. These findings were corroborated by correlative assessments with laser Doppler flowmetry and photoplethysmography [7, 26]. Unlike thermal challenges that rely on external heating or cooling [32], PBM interacts directly with cellular elements providing a physiologically relevant probe of microvascular function. In the current study, we apply this dynamic thermal imaging approach to leg ulcers by using PBM to stimulate regional microvascular flow, monitor the response, and evaluate outcome.

We hypothesize that DFUs respond favorably to PBM because of improved microvascular perfusion; whereas the response of VUs is poor since the prominent underlying pathology is increased hydrostatic pressure due to venous valvular insufficiency.

This study was therefore designed using dynamic thermal imaging to compare the changes in local skin temperature of DFUs and VUs induced by PBM as an indirect indicator of microvascular perfusion. Characterization here of the acute effects of PBM on microvascular flow in DFUs versus VUs using dynamic thermal imaging provides important new insight into the physiological pathway by which PBM supports repair and for distinguishing between wounds likely, and those less likely, to respond to PBM therapy.

## Materials and methods

### Study design

**Fig. 1:**
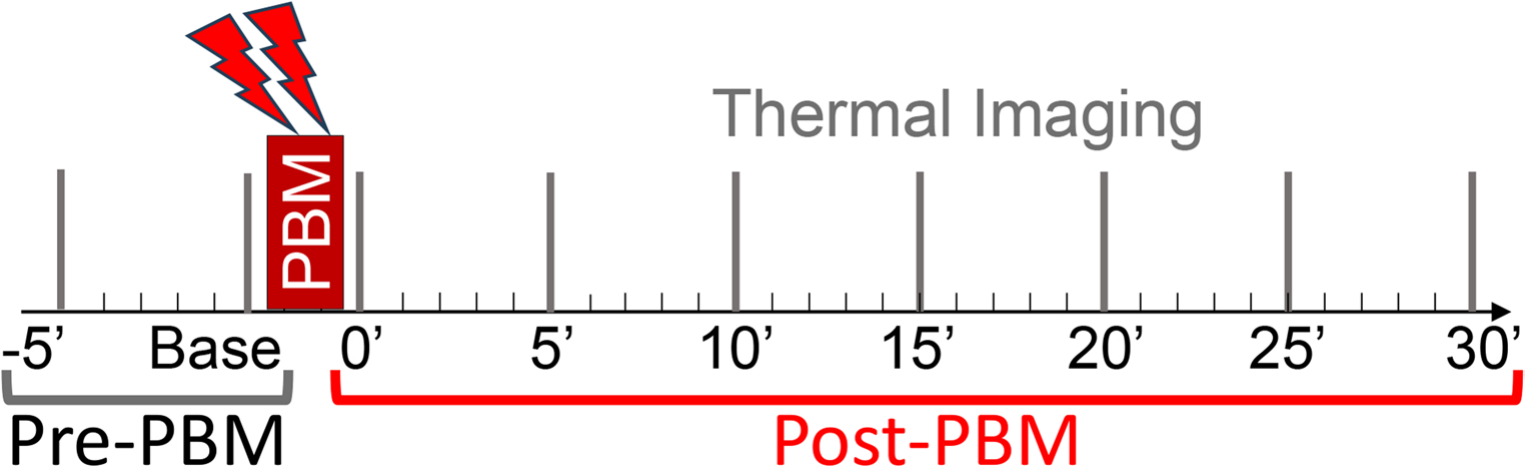
Study design. Timeline for photobiomodulation (PBM) treatment and dynamic thermal imaging. Two baseline thermal measurements (Pre-PBM) were acquired prior to PBM treatment, which was targeted at the ulcer bed, ulcer margins, and regional lymph nodes (indicated by the red bar and lightning symbol). Thermal images (timing indicated by grey lines) were acquired every 5 min over a 30-minute period (Post-PBM) to assess changes in surface temperature representing the microvascular blood flow

This is a prospective, controlled, diagnostic clinical trial. Patients with chronic DFUs or VUs, scheduled for regular wound treatment visits, were recruited at the outpatient wound clinic of the Scarborough Health Network, Toronto, Canada. Patient demographics and ulcer-related data (type, measurements, history, treatments) were collected. After dressing removal, ulcers were gently cleansed. Near-infrared PBM (see below) was applied to the wound bed, margins, and regional lymph nodes according to the regular PBM treatment protocol used at the clinic. Thermal images of the ulcer region were captured prior to PBM treatment and every 5 minutes (min) thereafter for 30 min post-PBM (Fig. 1). No visible exudate was present at the time of treatment or imaging. Minimum and maximum temperatures were automatically measured from the thermal images. The primary outcome was the change in wound temperature in response to PBM. Secondary outcomes included the temperature difference between the wound bed and its margins and correlations between the thermal response to PBM and wound characteristics.

### Ethics

The study design was approved by Scarborough Health Network Research Ethics Board. Since the study met the criteria for a quality improvement initiative under Tri Council Policy Statement: Ethical Conduct for Research Involving Humans (TCPS2) guidelines, informed consent was waived (determination dated April 16, 2021). Clinical trial number is not applicable.

### Study population and setting

Patients with chronic leg ulcers who visited the outpatient wound clinic at the Scarborough Health Network, Toronto, Canada were recruited between April 2021 to March 2022.

Inclusion Criteria were: (1) Chronic leg ulcers present for at least 3 months, (2) Ulcers located below the knee, and (3) Diabetic foot ulcer or venous leg ulcer. Exclusion Criteria were: (1) Ulcers resulting from surgery or cancer, (2) Patients undergoing active oncological treatment, and (3) Technically inadequate thermal images.

### Ulcer type

Ulcer type was determined prior to participation in the experiment, according to routine clinical guidelines, by a nurse practitioner (RR) specializing in wound care. In brief, DFUs are diagnosed by assessing neuropathy, peripheral arterial disease, and wound characteristics in diabetic patients; while VUs are identified through a history of chronic venous insufficiency supported by duplex ultrasound with distinct wound locations and appearances guiding differentiation [33, 34].

### Data collection

Demographic information, ulcer type, and relevant comorbidities were collected for all participants. Ulcer location was recorded as “non-foot” (ankle or shin) or “foot” (right/left and dorsal/plantar). Foot ulcers were further categorized as forefoot, midfoot, or hindfoot.

### Photobiomodulation

Ulcers were irradiated by a near-infrared GaAlAs PBM laser (B-Cure Laser Pro; Erica B-Cure Laser Ltd., Haifa, Israel), commercially available for over-the-counter use without prescription, that is part of the treatments regularly administered at the wound clinic. The energetic parameters include: 808 nm wavelength, peak power output of 250 mW, pulsation of 15 kHz with a 33% duty cycle, and spot size of 4.5 × 1.0 cm (4.5 cm²). The corresponding peak and average power densities are approximately 55 mW/cm² and 18 mW/cm² respectively. The treatment was applied continuously over the ulcer bed (distance of less than 1 cm) and in contact with ulcer margins for 0.5 min and 2 min respectively, repeated until full coverage according to the ulcer size. In addition, irradiation was administered in contact with the skin over the regional lymph nodes (popliteal and inguinal) for 2 min each.

### Dynamic thermal imaging

Prior to imaging, the ulcer area was gently cleansed and exposed to ambient room conditions for 10 min to allow the surface temperature equilibriate. Infrared-opaque stickers were placed around the ulcer to delineate the wound borders for optical-thermal registration. A 10-cm scale sticker was placed on the same plane as the ulcer to enable subsequent wound area measurements.

Thermal images were captured using a FLIR C5 infrared camera (FLIR Systems, Wilsonville, OR), mounted on a tripod at a fixed distance of 30 cm from the ulcer. Camera positioning was consistent as per ulcer location during the image session. Room temperature was recorded at the time of each image acquisition and entered into the FLIRTools software for calibration of temperature measurements. Room temperature during DFU imaging sessions was 24.8 ± 1.8 °C and during VU sessions 25.5 ± 1.6 °C. Two baseline images were captured before PBM treatment followed by six images post-treatment at 5-minute intervals up to 30 min. A total of eight images were obtained per ulcer (Fig. 1).

Thermal images were visualized using FLIR Tools software with a fixed temperature scale spanning 7 °C, using identical minimum and maximum temperature values across all time points. The maximum temperature for each subject was the highest temperature detected within that subject’s image series. A linear temperature color distribution was applied to enable meaningful visual comparisons of temperature changes over time. This process was performed separately for each subject in view of individual differences in baseline skin temperatures. The region of interest (ROI), defined by the infrared-opaque stickers, encompassed the ulcer bed and its margins. The software automatically identified the minimum and maximum temperatures within each ROI. Consistent anatomical marking of the ROI in relation to the infrared-opaque stickers was maintained across all time points. The exact positioning of the ROI was cross validated.

The PBM procedure combined with thermal imaging was performed prior to any other planned procedure. These included wound debridement that elicits tissue injury including bleeding and adjunctive therapeutic procedures such as hyperbaric oxygen, negative pressure wound therapy (PUSH), and compression therapy.

### Ulcer area measurement

The ulcer area was measured using ImageJ software. Boundaries were marked manually with the scale embedded in the image for calibration. Ulcer area was reported in cm².

### Statistical methods

#### Statistics

Continuous variables were summarized as means and standard deviations. Categorical variables were reported as frequencies and percentages. Repeated evaluations from the same patient were treated as independent samples when performed at separate visits, several weeks apart assuming clinically relevant variation and non-overlapping measurement conditions. To address potential concerns of statistical clustering, we performed sensitivity analysis for direction and magnitude, including only a single data point per patient from the first visit. Paired t-tests were used to compare temperatures between the 2 baseline images of each patient, before and after PBM challenge, and between ulcer beds and margins. Two-sample t-tests were applied to compare baseline temperatures and temperature changes between DFUs and VUs after Levin’s test for equality of variances. Pearson’s correlation coefficient was used to evaluate associations between baseline values and thermal response (‘baseline dependence’). Multiple linear regression was used to assess whether baseline wound temperature and wound area independently predicted the thermal response to PBM with separate models constructed for DFUs and VUs.

#### Power analysis

Assuming at least 10 ulcers of each type, and based on preliminary measurements of temperature change from baseline to 15 min post-PBM (diabetic ulcers: 2.0 ± 1.3 °C; venous ulcers: − 1.9 ± 3.2 °C), the study had 90.8% power to detect a statistically significant difference at a 5% significance level.

## Results

### Accountability and demographics

Twenty-five patients with chronic leg ulcers were assessed for eligibility of whom five were excluded: three had wounds located above the knee, and two had either post-surgical ulcers or were active oncology patients. The remaining 20 patients (13 males, 7 females) were included in the analysis. Of these, 13 had DFUs, primarily located on the forefoot, and 7 had VUs, predominantly on the shin or ankle (Table 1).

**Table 1:** Patient characteristics

Characteristic	DFU (*n* = 13)	VU (*n* = 7)
Age (mean ± SD), years	61.1 ± 17.8	76.3 ± 6.7
Sex (Male: Female)	10:3	3:4
Location – leg (ankle/shin), n (%)	2 (15%)	6 (86%)
Location – foot, n (%)	11 (85%)	1 (14%)
Forefoot, n (%)	8/11 (73%)	1/1 (100%)
Midfoot, n (%)	2/11 (18%)	–
Hindfoot, n (%)	1/11 (9%)	–
Deformity, n (%)	2 (15%)	1 (14%)
Amputation, n (%)	3 (23%)	0 (0%)
Ulcer area [cm^2^]*, (mean ± SD)	8.3 ± 10.0	7.3 ± 9.1
Ambulatory status		
Independent, n (%)	5 (45%)	5 (71%)
Walker/cane, n (%)	5 (45%)	2 (29%)
Non-ambulatory, n (%)	1 (9%)	0 (0%)

DFU – diabetic foot ulcers; VU – venous ulcers

*Calculated per wound (DFU: *n* = 22; VU: *n* = 11)

Patients with DFUs were significantly younger than those with VUs (*p* = 0.014). However, the mean ulcer area across all wounds was 7.9 ± 9.1 cm² with no significant difference between DFUs and VUs (*p* = 0.758)(Table 1). Among the DFU group, two patients had foot deformities, and three had undergone amputation. Regarding mobility, half of the patients, 10 (50%), were independently ambulatory, 7 (35%) used a walker or cane, and one patient (5%) was non-ambulatory.

Standard care included wound debridement and various dressings (e.g., Hydrofera Blue, honey-based products). In addition, patients received adjunctive therapies where clinically indicated including: hyperbaric oxygen, PUSH, and compression therapy. Comorbidities included hypertension, hypercholesterolemia, and cardiovascular disease—conditions typical of this patient population. Five patients had wounds assessed at more than one-time point (see Statistical Analysis section for handling of repeated visits). Thus, a total of 33 wounds were evaluated, comprising 22 DFUs and 11 VUs.

### Temperature: wound bed vs. wound margins

**Fig. 2:**
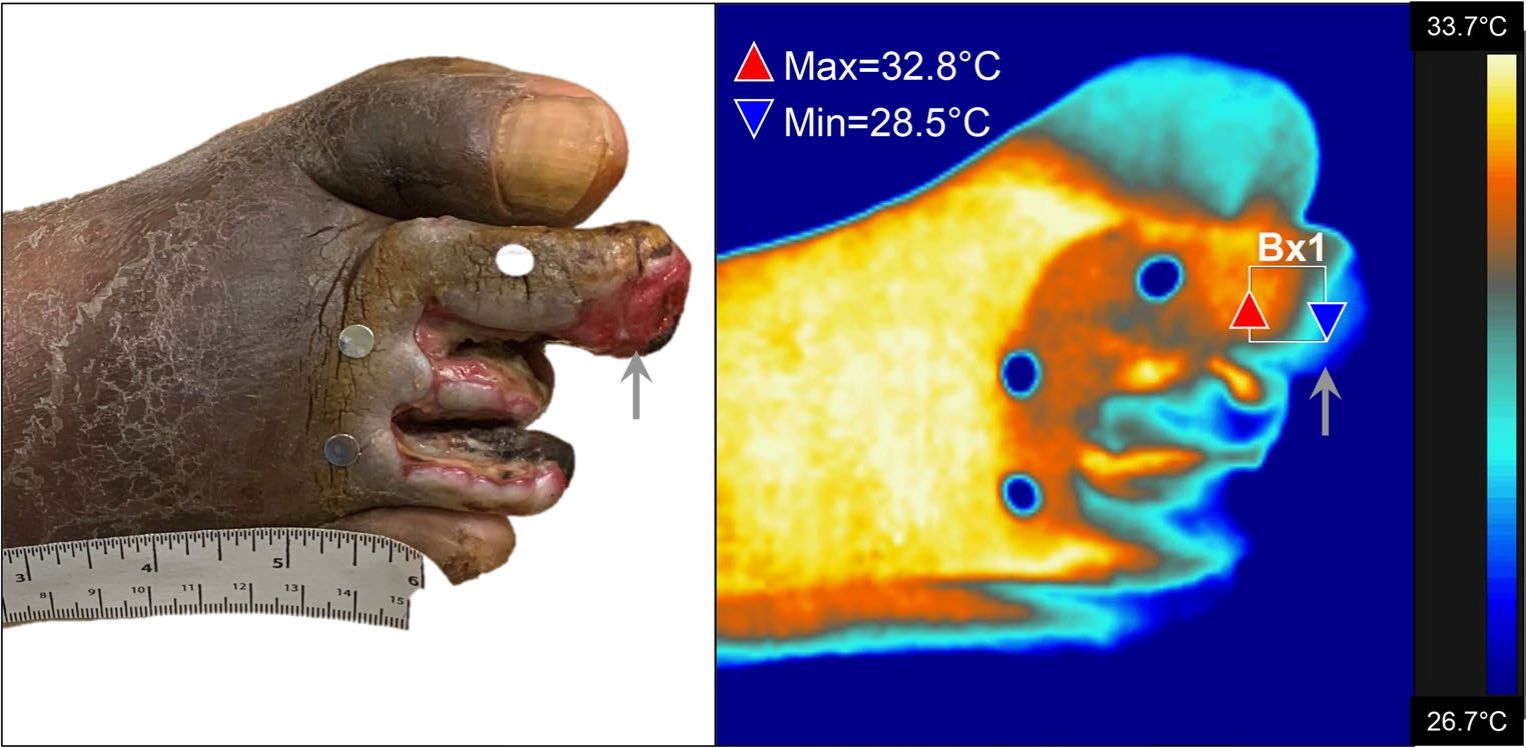
Thermal and clinical image of a Diabetic Foot Ulcer (DFU). Left: Chronic DFU with silver markers for identification of anatomical position. Right: Corresponding thermal image demonstrating reduced surface temperature at the ulcer site (grey arrow) compared to the surrounding tissue. Minimum temperature at center of ulcer = 28.5 °C; maximum temperature at the periphery = 32.8 °C. Bx1 denotes the region of interest used for temperature extraction. Thermal mapping reflects impaired microvascular flow in the wound bed relative to the margins

Using thermal imaging software, we found that all ulcers were significantly colder than the surrounding skin (*p* < 0.001) (Fig. 2). The absolute minimum temperature in the wound bed was 27.7 ± 2.9 °C, regardless of wound type (mean ± SD: VU = 28.0 ± 2.2 °C; DFU = 27.5 ± 3.2 °C; *p* = 0.878 by repeated measures ANOVA). Furthermore, the wound bed was, on average, 4.6 ± 2.2 °C colder than the adjacent wound margin.

### Dynamic thermal imaging

No significant difference was observed between the 2 thermal images taken Pre-PBM at time points − 5 and Base, ruling out environmental confounders as an explanation for subsequent temperature change (mean ± SD, T-5 vs. T_Base: 27.8 ± 2.9 °C vs. 27.3 ± 3.2 °C, 0.147 by paired ttest).

**Fig. 3:**
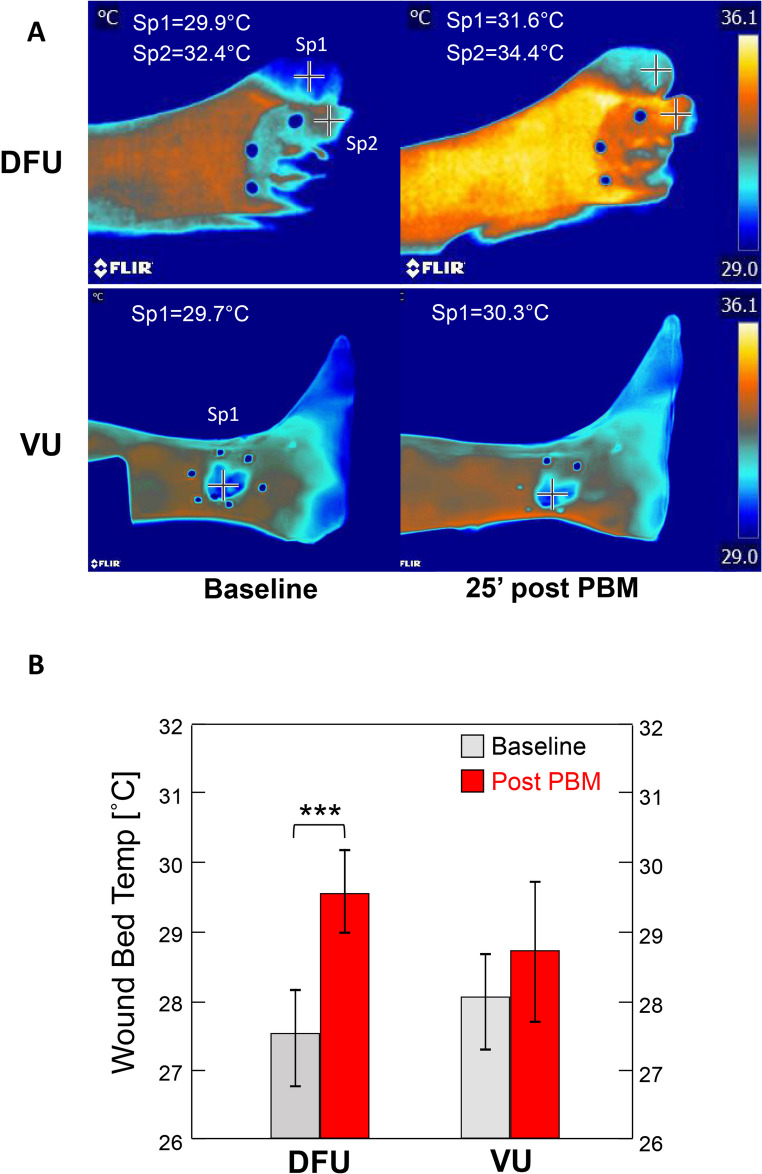
Thermal images of photobiomodulation treatment of diabetic versus venous ulcers. (**A**) Thermal images of a diabetic foot ulcer (DFU, top row) and a venous ulcer (VU, bottom row) before (Baseline [left]) and 25 min after PBM treatment [right]. Note that the surface temperature of the DFU increased both at the peri-wound site (Sp2: 32.4 °C → 34.4 °C) and the adjacent intact toe (Sp1: 29.9 °C → 31.6 °C)-- consistent with improved microvascular perfusion. In contrast, the VU showed minimal change following PBM (Sp1: 29.7 °C → 30.3 °C). A consistent thermal scale (29.0–36.1 °C) is used across all panels for comparability. (**B**) Bar graphs showing the wound bed temperature (mean ± SEM) before (baseline) and after PBM. Data represent the average temperatures over 10–30 min. for DFUs (n = 22) and VUs (n = 11). ***p < 0.001 by paired t-test

PBM challenge resulted in a significant increase in the wound bed temperature from baseline of DFUs but not of the VUs (Temp change from baseline [°C], mean ± SD: DFU [*n* = 22] = 2.1 ± 2.2, *p* < 0.001; VU [*n* = 11] = 0.7 ± 2.5, *p* = 0.385, by paired t-test) (Fig. 3). Sensitivity analysis, after excluding data from patients that contributed multiple data points, did not affect the effect magnitude or direction of the result (DFU [*n* = 13] = 2.2 ± 2.2, *p* = 0.003, VU [*n* = 7] = 0.5 ± 2.5, *p* = 0.638).

By multiple linear regression, the baseline wound temperature of DFUs strongly predicted the thermal response to PBM (β = − 0.521, *p* = 0.008); however, wound area was not predictive of this response (β = − 0.069, *p* = 0.143). The model explained 40% of the variance (R² = 0.399). In contrast, the same analysis performed on VUs showed that neither baseline wound temperature nor wound area were predictive of thermal response to PBM (model *p* > 0.05).

## Discussion

Among individuals with diabetes, 34% develop DFUs, which are associated with a markedly increased risk of amputation and mortality [2]. Peripheral neuropathy, commonly associated with DFU formation, affects nearly half of diabetic patients, especially in Western countries [35] and leads to a loss of protective sensation. Compromise of microvascular flow to the skin and cutaneous nerves by vasa nervorum contributes to its development. The longest nerve fibers are often affected first which is reflected in its frequent initial manifestation in the feet [35]. As a result, trauma- and pressure-prone areas, such as the tips of the toes and the medial side of the first metatarsal, are especially vulnerable to ulceration [36].

VUs are the most common type of leg ulcer accounting for approximately 80% of cases [37]. VUs typically occur in the gaiter area of the lower leg -- particularly medially. The principal etiology is venous valvular insufficiency leading to increased retrograde flow, abnormally elevated hydrostatic pressure, and capillary leakage with its large spectrum of local dermatologic and systemic consequences including stasis and pigmentation changes of the skin, impaired oxygen and nutrient delivery to tissues, inflammatory cell infiltration with or without superimposed infection, varicosities, thrombosis, and pain often leading to impaired mobility and reduced quality of life [38].

This study evaluated the thermal response of chronic leg ulcers to PBM using dynamic thermal imaging as a proxy for microvascular perfusion. We found that both DFUs and VUs were colder initially than surrounding skin -- consistent with impaired perfusion [39]. PBM therapy resulted in a significant and sustained increase in temperature in DFUs but not in VUs, and the response in DFUs was strongly influenced by baseline wound temperature with colder DFUs showing greater improvement. These findings demonstrate that dynamic thermal imaging coupled with PBM can capture physiologically relevant perfusion responses providing useful, practical assessment of microvascular function. While the DFU group was slightly older than the VU group, the difference in mean age was modest within an older population and the comparison primarily reflects ulcers with distinct pathophysiological mechanisms affecting the microcirculation. Although healing outcomes were not assessed, the baseline-dependent response in DFUs suggests that PBM may be particularly effective for poorly perfused tissues, and that this approach could help identify DFUs most likely to benefit from this microvascular-stimulating treatment approach providing for optimization of therapeutic protocols.

The wound bed temperature at baseline (prior to PBM) was 27.6 °C and not significantly different between DFUs and VUs. This is 4.6 °C colder than the temperature of the surrounding intact skin and well above the 0.5 °C threshold considered clinically meaningful [40, 41] indicating markedly impaired perfusion at the area of the wound bed. Mendonça et al. [42] reported a difference of 2.9 ± 2.2 °C between the wound bed and normal skin in VUs, while Nam et al. [43] described a 4.0 ± 0.1 °C difference between a DFU-affected foot and the contralateral foot in patients with peripheral artery disease. Note that because thermal imaging captures superficial skin temperature, the measured signal primarily reflects microvascular perfusion within the dermal capillary network [7], whereas peripheral arterial disease mainly affects deeper macrovascular inflow.

Some studies monitored skin temperature to predict ulcer formation noting that increased temperatures can precede ulceration [44, 45]. It has been suggested that neuropathic DFUs can present as being warmer than surrounding skin [42]. Although the neuropathy status was not assessed in the current study, none of the DFUs in our cohort presented with a warmer wound bed than the adjacent skin. Our study focused on established ulcers. Nonetheless, this emphasizes the importance of identifying secondary and co-existing pathologies in order to maximize treatment outcome.

The primary objective of the current study was to characterize the thermal response to PBM of DFUs and VUs. Wound bed temperatures were recorded immediately after PBM and every 5 min for 30 min. Because local heating effects typically dissipate within 10 min [32], only the 10–30 min post-PBM measurements were analyzed excluding the immediate and 5-minute time points to avoid possible confounding effects in cases of initial photothermal heating.

In DFUs, skin temperature over the wound bed and surrounding tissue increased significantly after PBM and remained elevated for at least 30 min. This sustained rise is consistent with enhanced microvascular blood flow as reflected by thermal imaging. Baseline wound temperature was a significant independent predictor of this response, explaining ~ 40% of the variance, whereas wound size showed no association. This is notable given that ulcer area ≥ 1 cm² has been identified as an independent risk factor for increased mortality in DFU patients (hazard ratio 1.50; 95% CI 1.42–1.59) [46]. The lack of association in our study suggests that vascular status, rather than ulcer extent, may be more critical in determining physiological responsiveness to PBM. Indeed, previous clinical trials have reported benefits of PBM even with very large DFUs [28] reinforcing the view that perfusion status, rather than size alone, determines therapeutic potential. The moderate negative correlation between baseline temperature and PBM response further indicates that colder DFUs—reflecting poorer perfusion—benefit most from PBM-induced vasodilation.

By contrast, no significant temperature change was observed in VUs treated by the same PBM protocol. This likely reflects the distinct difference in pathophysiologies of the two ulcer types. However, the lack of response in VUs may reflect protocol-specific effects such as alternative wavelengths, administration techniques, or dosing strategies. We suggest that the PBM-induced rise in temperature in DFUs is consistent with enhanced microvascular perfusion, while the minimal net change in temperature by PBM in VUs is due, at least in part, to initial redistribution of pooled blood.

The findings of the current study emphasize the need for patient-specific PBM protocols taking into account the differences in etiology and pathogenesis of the various types of ulcers, as well as possible co-existing pathologies, in order to provide effective therapy.

### Limitations

This study was not controlled for the use of vasoactive medications or for neuropathy status in patients with diabetic foot ulcers, both of which may influence microvascular responses, and it did not include a sham irradiation control. Environmental conditions were mitigated by obtaining two baseline thermal images prior to treatment and by recording ambient room temperature for calibration of thermal measurements, while analyses were adjusted for baseline temperature reflecting the underlying perfusion status of the wound. Some patients contributed more than one observation; however, sensitivity analysis restricted to one observation per patient yielded similar results. Future studies with larger cohorts and inclusion of a sham control group will further clarify the physiological and clinical implications of the observed thermal responses.

### Conclusions

Whereas enhanced microvascular flow is central to the beneficial effects of PBM in diabetic foot ulcers, PBM did not produce comparable response in venous ulcers, the principal pathogenesis of which is increased hydrostatic pressure due to venous valvular insufficiency. This study demonstrates the feasibility of coupling PBM with dynamic thermal imaging for use as a novel diagnostic tool for chronic ulcers and for the assessment of acute responsiveness to PBM protocols on a patient-specific basis.

## Data Availability

The data that support the findings of this study are available on request from the corresponding author. The data are not publicly available due to privacy or ethical restrictions, as the clinical dataset contains coded but potentially re-identifiable patient information, and public sharing could compromise participant confidentiality.

## Declarations

### Patient involvement

Study participants were not involved in the design, conduct, interpretation, or translation of the current research.

## Abbreviations

DFU: Diabetic Foot Ulcer
PBM: Photobiomodulation
ROI: Region of Interest
VU: Venous Ulcer
